# Phosphorylation of plant virus proteins: Analysis methods and biological functions

**DOI:** 10.3389/fmicb.2022.935735

**Published:** 2022-07-26

**Authors:** Xinjian Zhuang, Xiao Guo, Tianxiao Gu, Xiaowei Xu, Lang Qin, Kai Xu, Zhen He, Kun Zhang

**Affiliations:** ^1^Department of Plant Protection, College of Horticulture and Plant Protection, Yangzhou University, Yangzhou, China; ^2^Jiangsu Key Laboratory for Microbes and Functional Genomics, Jiangsu Engineering and Technology Research Center for Microbiology, College of Life Sciences, Nanjing Normal University, Nanjing, China

**Keywords:** phosphorylation, replication, intracellular movement, long-distance movement, infection cycle

## Abstract

Phosphorylation is one of the most extensively investigated post-translational modifications that orchestrate a variety of cellular signal transduction processes. The phosphorylation of virus-encoded proteins plays an important regulatory role in the infection cycle of such viruses in plants. In recent years, molecular mechanisms underlying the phosphorylation of plant viral proteins have been widely studied. Based on recent publications, our study summarizes the phosphorylation analyses of plant viral proteins and categorizes their effects on biological functions according to the viral life cycle. This review provides a theoretical basis for elucidating the molecular mechanisms of viral infection. Furthermore, it deepens our understanding of the biological functions of phosphorylation in the interactions between plants and viruses.

## Introduction

**FIGURE 1:**
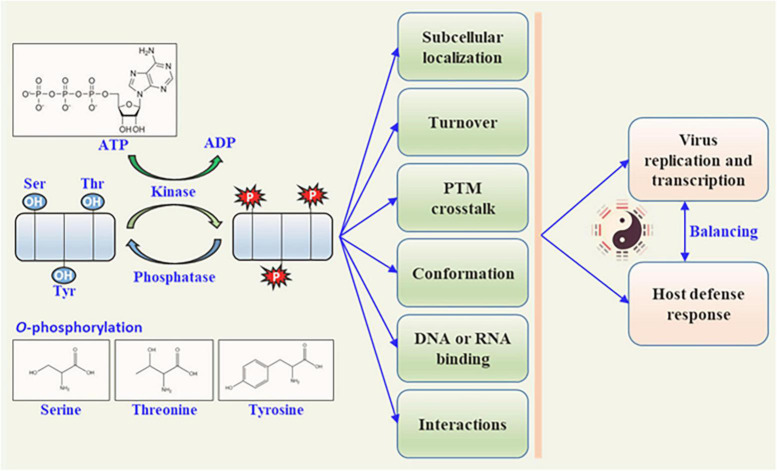
Biological function of plant viral protein phosphorylation. Protein kinases phosphorylate plant viral proteins to regulate the viral protein activity by phosphorylation and dephosphorylation (catalyzed by phosphatases). The functions of viral proteins are altered in different ways, including subcellular location, pathogenicity, intrinsic biological activity, DNA or RNA binding activity, self-interaction, and interaction with other proteins. These functional changes in plant viral proteins further affect viral replication or transcription, and the host defense responses.

Rapid responses to internal and external cues are vital for complex life on the earth. Whether to maintain optimal conditions for growth or to avoid biotic/abiotic stresses, these responses must occur on a timescale that affords the organisms an obvious survival advantage. Cells have evolved sophisticated regulatory systems that can sense, transmit, sort, store, and interpret information that could enact a coordinated and timely response to the changes in environments. Post-translational modification (PTM) is an elegant transducer of the signals for adaptations of living organisms by the reversible and rapid nature, relatively small expenses, and profoundly function modulation of the target protein. Examples of PTM include phosphorylation (Ramazi and Zahiri, 2021), glycosylation (Vigerust and Shepherd, 2007), ubiquitination (Shen et al., 2016), acetylation (Diallo et al., 2019), lipidization (Chiang et al., 2013), small ubiquitin-like modifier (SUMOylation) (Cheng et al., 2017), and lysine succinylation (Zhang et al., 2011), butyrylation (Chen et al., 2007), or crotonylation (Wan et al., 2019). Phosphorylation is the most intensively investigated PTM and is involved in almost every cellular process (Cohen, 2002; Duan and Walther, 2015). Numerous metabolic enzymes, such as pyruvate dehydrogenase and glycogen synthase, occur in the phosphorylation PTM (Fischer and Krebs, 1955). The mechanisms by which phosphorylation controls protein functions include modulation of protein–protein interactions and changes in protein conformation. Phosphorylation often functions as a docking site for intra- or inter-molecular protein interactions via protein conformation changes, and directly modulates enzymatic activity, regulates subcellular localization, targets turnover, and affects signaling transduction by other PTMs (Figure 1). Overall, the phosphorylation by kinases and de-phosphorylation by phosphatases serve as low-cost and high-efficiency molecular regulators that can alter the behavior of the targets directly or indirectly.

There are four types of phosphorylations, the first being *O*-phosphorylation, which mainly occurs at the hydroxyl residues of serine (Ser), threonine (Thr), tyrosine (Tyr), and hydroxyproline (Emmer et al., 2013). The second is *N*-phosphorylation, which occurs on histidine and lysine amino residues (Ni et al., 2009). The third is *S*-phosphorylation, which occurs in the sulfhydryl group of cysteine (Buchowiecka, 2014). The last type is *Acyl*-phosphorylation, which is mainly observed in the acyl groups of aspartic acid and glutamate (Cheng et al., 1996). *O*-phosphorylation is the most common type of phosphorylation in eukaryotes (Panni, 2019). Heibeck et al. (2009) showed that the relative abundance ratio of three phosphorylated amino acids (AA) (pSer : pThr : pTyr) in a single cell is 1800 : 200 : 1, indicating that Ser phosphorylation is the most common modification (Heibeck et al., 2009). In eukaryotes, *O*-phosphorylation is catalyzed by protein kinases, which function by adding the γ-phosphate group of ATP or GTP to the hydroxyl side chain of Ser, Thr, Tyr, and hydroxyproline (Krupa et al., 2004). Different protein kinases can recognize and modify different sites of the target protein. Phosphorylated proteins can also be dephosphorylated by phosphatases, which reduce the phosphate group from the side chain of the target protein (Millar et al., 2019). These dynamic and reversible PTM changes are energy-consuming processes that require coordination between the protein kinase and phosphatase to regulate the folding style and conformation of the target protein (Jakubiec and Jupin, 2007). The reversible processes of phosphorylation and dephosphorylation act as an “ON-OFF SWITCH”-like modulator of their functions, such as regulating enzymatic activity, protein stability, protein virulence, subcellular localization of target proteins, signaling transduction and recognition, and interaction with other proteins (Friso and van Wijk, 2015).

In plant virology, studies on the phosphorylation modification of viral proteins have recently progressed. The regulatory function of phosphorylation is involved in most steps of the plant viral life cycle, including protein translation (Martínez-Turiño et al., 2018), replication of viral genomic materials (Gao et al., 2020), intercellular movement (Nemes et al., 2017), cell-to-cell movement (Hung et al., 2014), virion assembly (Nemes et al., 2019), and long-distance movement (Zhao et al., 2015). Although several viral proteins have been extensively studied, the molecular mechanisms of their action in viral infection have not been elucidated. A lack of unified, standard, and high-efficiency phosphorylation detection methods and detailed phosphorylation site identification still remains. In addition, the biological functions of phosphorylated viral proteins are distinct and independent for each plant virus studied. In this review, the functional phosphorylation analyses that were not within the scope of the plant virus life cycle and, accordingly, the relevant studies have not been included.

A clear understanding of the phosphorylation status of plant viral proteins in different environments would be helpful to further analyze the biological functions of phosphorylation. Hence, summarizing the methods for identifying the phosphorylation of plant viral proteins and the biological functions of phosphorylated plant viral proteins is urgently needed to gain a deeper understanding of the multiple functions of viral proteins during infection. This study aims to provide a reference for further study of the molecular mechanisms of plant viral protein phosphorylation and underlines the significance of the PTM in virus infection cycles.

## Common assays for identification of phosphorylated plant viral proteins

The online services, such as Kinasephos^1^ and Netphos 3.1^2^, can accurately predict the phosphorylation sites of plant virus proteins and the corresponding kinases involved (Zhang et al., 2018). Furthermore, various methods, including liquid chromatography-mass spectrometry (LC-MS/MS) (Gao et al., 2020) and radioisotope labeling ([γ-^32^P]ATP) experiments *in vivo* and *in vitro*, have been widely used for the identification of viral protein phosphorylation (Hu et al., 2015). Selective protein kinase inhibitor screening (Zhang et al., 2018), commercial pSer/pThr/pTyr phosphorylation-specific antibodies (Li et al., 2022), and commercial Phos-tag™ gel-based immunoblotting have been widely used for the identification of the phosphorylation of viral proteins. Here, we summarize the recently published phosphorylation identification assays for plant viral proteins, which include identification of the phosphorylation of target proteins and the detailed phosphorylation sites on target proteins, in order to provide a basis and reference for further studies.

### Liquid chromatography-mass spectrometry-based identification of phosphorylated plant viral proteins

The continuous and rapid development of mass spectrometry and computer-based bioinformatics have improved the feasibility of research on viral protein PTMs and the interactions between plant viruses and hosts. Trypsinized protein samples are ionized and analyzed via mass spectrometry using the time-of-flight under an electric or magnetic field (Domon and Aebersold, 2006). The detector reports the relative intensity and mass-to-charge ratio of each peptide or signal. Based on the extra 79.966 KDa difference between the phosphorylated and unphosphorylated peptides under the condition of one potential phosphorylation site, AA sequences of the target peptide, and other fragmented ion signals from secondary mass spectrometry, phosphorylation can be easily identified with the bioinformatics software available nowadays (Zhang G. et al., 2014).

Various strategies have emerged for enriching phosphorylated peptides or proteins to improve the sensitivity and validity of phosphorylation assays. These approaches increase the abundance of phosphorylated peptides or proteins in LC-MS/MS assays. These enrichment methods include pSer/pThr/pTyr phosphorylation-specific antibody-based affinity capture (Stoevesandt and Taussig, 2013), chemical derivatization of phosphorylated AAs (Deng et al., 2007), metal ion-based affinity capture (Blacken et al., 2008), ion-exchange chromatography (Jungbauer and Hahn, 2009), and classical immunoprecipitation technology using phosphorylation-specific antibodies (Kurosaki et al., 2019). The enriched proteins are hydrolyzed to form peptides via trypsin digestion *in vitro*, and LC-MS/MS analyses are performed to detect the phosphorylated sites. Various phosphorylation sites can be identified using conventional LC-MS/MS analysis combined with the appropriate software. However, it is not sufficient to show that the target protein is phosphorylated, and additional phosphorylation analyses are necessary to further confirm the results.

### Radioisotope (^32^P) labeling of plant viral proteins *in vitro*

Under kinase catalysis, the radioisotope ^32^P-labeled ATP that acts as an energy source (γ-position phosphate group) is transferred to the substrate protein by kinase (Lamberti et al., 2011). In the study by Lamberti et al. (2011), the corresponding proteins were separated via sodium dodecyl sulfate polyacrylamide gel electrophoresis (SDS-PAGE) after *in vitro* catalytic reactions were completed. The gel was dried using a Model 583 Gel Dryer (Bio-Rad, Hercules, CA, United States), and the phosphorylated proteins were detected using X-autoradiography or a phosphorous storage screen (BAS-IP TR 2040 E Tritium Screen, GE Healthcare, Chicago, IL, United States). This method is the most direct protein phosphorylation detection assay *in vitro*. It has the advantages of high sensitivity and accuracy and has been widely applied in the identification of plant viral protein phosphorylation. Using this method, the phosphorylation of a single protein was proven *in vitro*, and the detailed AA sequences of the substrate protein and catalytic kinase were validated. However, this is a hazardous procedure for operators, especially for first-time and inexperienced researchers.

The specific steps for *in vitro* phosphorylation experiment are as follows. A 20 μL phosphorylation reaction system is prepared with 20 mmol.L^–1^ Tris-HCl buffer (pH = 7.5), 0.1 μg kinase/total protein extractions, 1.0 μg substrate (purified protein), 25 μmol.L^–1^ ATP, 1 μCi [γ-^32^P]-ATP (China Isotope & Radiation Corporation, Beijing, China), 5 mmol.L^–1^ MgCl_2_ (or MnCl_2_, CaCl_2_, etc.), and ultrapure water. After incubation at 30°C for 30 min, 5 μL of 2 × SDS loading buffer [100 mM Tris–HCl, pH = 6.8, 4% SDS (v/v), 20% glycerol (v/v), 0.2% bromophenol blue (v/v), and 5% β-mercaptoethanol (v/v)] are added. The reaction is terminated by denaturation at 95°C for 5 min. All proteins in the reaction system are then separated via 12.5% SDS-PAGE for 2 h at 80 V. The gel is placed on a filter paper (8 cm × 10 cm), and dried in a gel dryer for 40 min. The dried gel is exposed to X-rays and detected using a phosphorous storage screen or film (Kodak X-OMAT BT, Nanjing, China) (Hu et al., 2015). Specific antibodies or phosphorylated antibodies can also be used to detect the target substrate proteins via western blotting.

### Phos-tag™ sodium dodecyl sulfate polyacrylamide gel electrophoresis for identification of phosphorylated plant viral protein

Phos-tag™ reagent can specifically bind to the phosphate group on the phosphorylated protein after binding with Mn^2+^ or Zn^2+^ to form a relatively stable compound (Kinoshita-Kikuta et al., 2014). When Mn^2+^ or Zn^2+^ and Phos-tag™ are added to a conventional SDS-polyacrylamide gel, the phosphorylated protein binds to the Phos-tag™. It forms a larger complex than the unphosphorylated protein, leading to a significant reduction in the electrophoretic migration rate of the phosphorylated protein in the gel under electrophoretic conditions (Kinoshita et al., 2006). Therefore, the Phos-tag™ reagent can specifically separate the phosphorylated proteins from non-phosphorylated proteins. Specific or phosphorylated antibodies are usually combined with a Phos-tag™ for western blotting.

This simple method can be used to detect all types of phosphorylation *in vivo* and *in vitro*. The Phos-tag™ reagent in SDS-polyacrylamide gel is generally twice the concentration used in most experiments, often at a concentration of 20–100 μM (Nagy et al., 2018). The optimal concentration of the Phos-tag™ reagent used for phosphorylation assays should be determined based on the actual characteristics of the specific protein in the experiment. Combined with the western blotting and LC-MS/MS, the Phos-tag™ reagent could be used for protein purification in some situations. However, it often has low accuracy in protein phosphorylation assays used for the preliminary determination of protein phosphorylation. Further identification of protein phosphorylation usually requires additional evidence, such as western blotting or LC-MS/MS.

### Western blotting for identification of phosphorylated plant viral proteins

The target protein or system containing the target protein is electrophoresed via SDS-PAGE at 80 V for 2 h and then transferred onto a piece of nitrocellulose membranes (Bio-Rad, United States). Specific antibodies with high affinity for the target protein or phosphorylated Ser/Thr/Tyr are used to detect the target protein (Mei et al., 2018a). Secondary antibodies are cross-linked with the horseradish peroxidase or alkaline phosphate. Based on the specific binding activity of the antibody to the corresponding antigen, only the phosphorylated target protein can be detected, as evidenced by the strong bands or signals at specific positions on the membrane (Li et al., 2022).

The western blot assay is relatively easy to perform and has no equipment or location restrictions. Antigen–antibody recognition of the phosphorylation site is specific and has high resolution in western blotting. It is often used for the validation of LC-MS/MS results in phosphorylation assays. However, this method is semi-quantitative, it is difficult to identify new or multiple phosphorylation sites in a single protein because of the problems in preparing the corresponding specific phosphorylation antibody.

## Common kinases that phosphorylate plant viral proteins

Phosphorylation/dephosphorylation is involved in multiple biological processes that regulate plant growth, development, and aging, such as plant biotic/abiotic stresses, hormone signal transduction, ion transportation, and material metabolism (Yin et al., 2018). Different kinases often mediate different types of phosphorylation in the target protein. An increasing number of studies indicate that phosphorylation plays an important role in regulating viral genome replication, viral intracellular/intercellular movement, viral gene transcription, virus infectivity to the host plant, defense and counter-defense of plant innate immunity, and virion assembly.

Understanding the recognition mechanisms and biological functions of kinases and their substrates is essential. The surrounding phosphorylated AAs (Tyr/Ser/Thr) can be specifically recognized by the modular phosphoprotein-binding domains of kinases, called p-motifs. van Wijk et al. (2014) have explored the certain kind of kinases that phosphoprotein of the p-motifs (van Wijk et al., 2014). Several algorithms, such as PTMphinder, Motif-x, and Motif-All, have been developed to predict the relatively conserved motifs in phosphopeptides, based on the concept of p-motifs. The obvious phosphorylation motifs were categorized into two major types: pS type and pT types. The pS type was clustered into seven clades, including glutamate-rich, glycine-rich, DS with acidic residues downstream or basic residues upstream, SP motif, pS with acid residues, and serine-rich pS motif. The pT types were also divided into seven clades, namely, aspartate-rich, glutamate-rich, proline-rich (2 X), arginine-rich (2 X), and lysine-rich pT (van Wijk et al., 2014). The acidic S-type motifs included S-[DE], S-E, S-X-[DE], and S-X-X-[DE], and the common basic S-type motifs comprised [RK]-X-X-S, R-S, and [RK]-X-S. T-P is the most common motif in the pT motif. Arginine was often present in the basic T-type motif ([RK]-X-X-T), while aspartate or glutamate was included in the acidic T-type motifs (X-T-X-[ED]). Annotation from the PhosPhAt 4.0 database and the known-target relationships were obtained for the p-motifs identified by Li et al. (2022) in common substrates of different kinase families. This analysis revealed that one-third of the identified p-motifs in the substrate of MAPK were T/S-P (Pitzschke, 2015; Rayapuram et al., 2018). CDKs are tightly associated substrates that contained the S-E, S-[DE], and S-X-[DE] motifs (Xi et al., 2021). The basic S-motif was over-represented in the substrates of SnRK1, SnRK2, SnRK3, and LRR-RK. In the T-motifs group, T-P was found in substrates of multiple kinase families, such as CKII, CDK, CDPK, SnRK1, and SnRK3 (Xi et al., 2021). The existing database and the corresponding relationship between substrates and kinases contribute to the prediction of target kinases that are capable of phosphorylating proteins containing the specific p-motifs.

**FIGURE 2:**
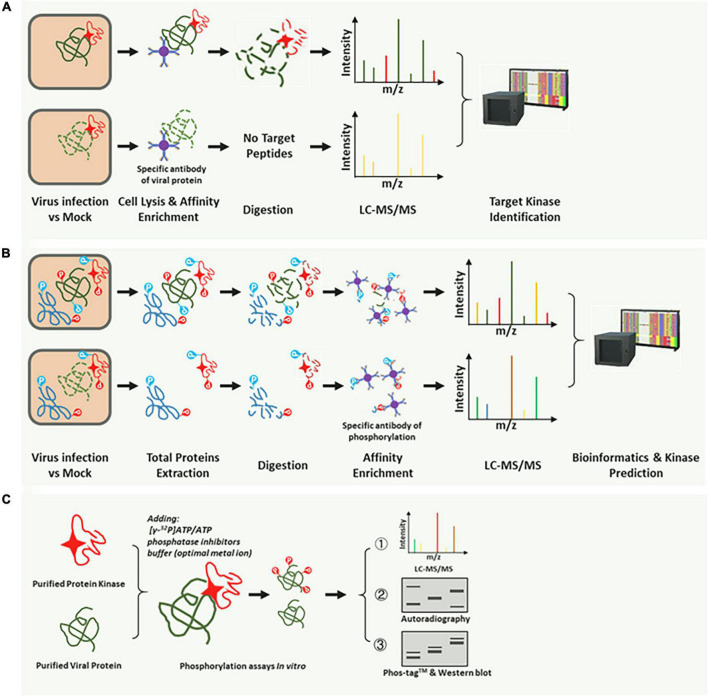
Common methods for identification of the corresponding kinase of the target substrate. **(A)** The target substrate interacts with and is phosphorylated by a specific kinase. A substrate-specific antibody was used for co-immunoprecipitation (Co-IP) of the corresponding kinase. The kinase could be identified by the LC-MS, and BLASTP was performed on the NCBI or the species-specific database. **(B)** High-throughput phosphoproteomic analysis based on the LC-MS/MS. The p-motifs could be determined using phosphor-peptides and interrogation of the corresponding database. Then, the kinase type could be identified using the already phosphorylation mode in the database. Using the third method (C), a specific kinase can be confirmed. **(C)** *In vitro* phosphorylation reaction system for further validation of the target kinase. Radioisotope (^32^P)-labeled ATP, Phos-tag™ SDS-PAGE, and pS/pT-specific antibodies were used in the reaction, and specific kinases were easily identified.

Rapid and directed identification of hundreds of p-motifs via advanced mass-spectrometry and faster bioinformatics techniques has become feasible (Amanchy et al., 2007). Several phosphorylation prediction databases based on these accumulated datasets have based customized retrieval (Gao et al., 2009; Cheng et al., 2014; Schulze et al., 2015; Millar et al., 2019), thereby allowing the prediction of a possible optimal kinases type. In this study, we summarized the identification methods used in published studies. According to the operations, these methods are divided into three types. The first method involves comparative LC-MS/MS and the kinase-interaction assay, which can identify the corresponding kinase that can phosphorylate and directly interact with the target substrate (Figure 2A; Gil et al., 2019). The second method is high-throughput phosphoproteomics analyses, which can be applied where the corresponding kinase can phosphorylate but not interact with the target substrate (Figure 2B; Poss et al., 2016). The third method is the use of *in vitro* methods for validation of phosphorylation (Figure 2C; Lipton et al., 2015). Generally, the phosphorylation reaction *in vitro* (Figure 2C) is often combined with the top two analysis methods (Figures 2A,B) to further validate the substrate and the corresponding kinase. An *in vitro* phosphorylation reaction system or a cell lysis reaction should contain a phosphatase inhibitor.

Previous investigations have shown that a variety of plant kinases can phosphorylate the proteins encoded by plant viruses, including the casein kinase family (CKs) (Martínez-Turiño et al., 2018), glycogen synthase kinase-3 (GSK-3) (Mei et al., 2018a), sucrose non-fermenting-1-related protein kinase (SnRK) (Shen et al., 2018), protein kinase A (PKA) (Makarov et al., 2012), and protein kinase C (PKC) (Samuilova et al., 2013). We have summarized these publications and listed the common motifs of phosphorylated substrates of these different protein kinases in Table 1. This summary will provide information to help identify common kinases in phosphorylation assays. In particular, by combining the *in vitro* and *in vivo* phosphorylation reaction experiments, the corresponding kinase or target phosphorylation site can be quickly confirmed.

**TABLE 1:** Consensus phosphorylation sites catalyzed by common plant kinases.

Protein kinase	Full name	Consensus of the phosphorylation site	References
AMPK	Adenosine 5‘-monophosphate (AMP)-activated protein kinase	B-(X, R/K/H)-X-X-S /T-X-X-X-B	Pinna and Ruzzene, 1996
CDK	Cyclin-dependent kinase	S/T-P-X-K/R	Pinna and Ruzzene, 1996
CKI	Casein kinase-1	pS-X-X-S/T	Flotow et al., 1990
CK2	Casein kinase-2	S/T-D/E-X-E/D	Meggio and Pinna, 2003
GSK3	Glycogen synthase kinase-3	S/T XXX pS/pT	Fiol et al., 1990
PKA	Protein kinase A	R-R-X-S/T-Φ	Songyang et al., 1994
PKB	Protein kinase B	R-X-R-X-X-S/T	Obata et al., 2000
PKC	Protein kinase C	X-R-X-X-S/T-X-R-X	Pinna and Ruzzene, 1996
PKD	Protein kinase D	L/I-X-R-X-X-S/T	Hutti et al., 2004

pS/pT, phosphorylated Ser/Thr; X, any residue except those which may play a negative role; Φ, hydrophobic residue; B stands for any hydrophobic amino acid.

### Casein kinase family

Protein kinase 1 (CK1) is a highly conserved serine/threonine kinase in eukaryotes. Its N-terminal catalytic domain is relatively conserved and functions in the recognition of substrate proteins (Gross and Anderson, 1998). The C-terminal domain of CK1 has low conservation in different species and is responsible for the binding specificity of different substrate proteins (Graves et al., 1993; Graves and Roach, 1995; Cegielska et al., 1998). CK1 has been widely studied in both yeasts and animals (Knippschild et al., 2005). To date, seven CK1 types have been identified in mammals that are involved in the regulation of vesicle transport, growth, development, morphological changes, DNA repair, and cytokinesis. CK1 participates in signal transduction and hormone metabolism in rice (Liu et al., 2003; Dai and Xue, 2010). In *Arabidopsis*, CK1 regulates blue-light signaling and ethylene biosynthesis through phosphorylation (Tan and Xue, 2014). Additionally, the *Arabidopsis* genome encodes at least 14 CK1-like (CKL) protein kinases located in the cytoplasm, nucleus, endoplasmic reticulum, and vesicular granular structure and participates in the regulation of multiple biological processes (Lee, 2009).

Protein kinase 2 (CK2) phosphorylates various plant viral proteins and plays an important role in the regulation of viral infection and host defense. CK2 is a highly conserved serine/threonine-specific kinase with multiple physiological functions in eukaryotes (Mulekar and Huq, 2014). The CK2 holoenzyme comprises two α-catalytic subunits and two β-regulatory subunits that constitute heterotetramers *in vivo* (Litchfield, 2003). In plants, the core CK2α catalytic subunits can play an independent role in the phosphorylation of target proteins (Filhol et al., 2004). Most CK2 proteins belong to a multigene family in plants. CK2 homologous genes have been cloned from various plants, including *Nicotiana attenuata*, wheat, corn, rice, and barley (Mulekar and Huq, 2014). CK2 is involved in the regulation of plant growth, development, biotic/abiotic stresses, light signals, and circadian rhythms (Huq, 2006; Moreno-Romero et al., 2008; Nagel and Kay, 2012; Mulekar and Huq, 2014). CK2-mediated phosphorylation of substrate proteins usually leads to changes in protein quaternary structure and conformation, which results in changes in DNA binding, RNA-binding, dimerization, stability, protein–protein interactions, and subcellular localization (Mulekar and Huq, 2014).

### Glycogen synthesis kinase-3

Glycogen synthesis kinase-3 (GSK3) is a serine/threonine phosphokinase that participates in all biological processes (Kim and Kimmel, 2000). In mammals, GSK3 regulates cell metabolism, signal transduction, embryonic development, and neuronal differentiation (Doble and Woodgett, 2003; Kim and Kimmel, 2006; Hur and Zhou, 2010; Wu and Pan, 2010). Most protein substrates of GSK3 contain a common phosphorylation motif (Ser/Thr-x-x-Ser/Thr) (Fiol et al., 1990; Pinna and Ruzzene, 1996; Table 1). Various GSK3-like kinases are also present in plants. Compared with animal GSK3, plant *GSK* genes are more diverse (Yoo et al., 2006). Phosphorylation of the C-terminal serine/threonine residue of GSK3 is catalyzed by another kinase, which promotes its catalytic activity in animals (Thomas et al., 1999). In plants, the GSK3-like kinases tend to interact directly with the substrate proteins and phosphorylate them. GSK3-like kinases play different roles in cell growth, root and stomatal cell development, flowering, and fruiting (Youn and Kim, 2015).

### Protein kinase A and protein kinase C

Protein kinase A (PKA) is an enzyme family whose catalytic activity depends on the levels of cyclic adenylate (cAMP) (Gold, 2019). It is a serine/threonine phosphokinase that is involved in the regulation of multiple biological processes, including cell proliferation and glycogen, and lipid metabolism (Endicott et al., 2012). PKA is a tetramer comprising two regulatory and catalytic subunits (Zelada et al., 2002). In eukaryotic cells, cAMP activates PKA, which in turn phosphorylates the target substrate proteins. The activated PKA catalytic subunit can phosphorylate the serine or threonine residues of target proteins in cells, leading to changes in the activity, which further affects the expression of related genes (Yang, 2018). Although PKA has not been identified in plants thus far, plant virologists have found that the PKA-like kinases, which phosphorylate plant viral proteins and regulate their functions in the viral life cycle, are common in plants.

Protein kinase C is also a serine/threonine phosphokinase, composed of an N-terminal regulatory region (approximately 20—40 kDa) and a C-terminal catalytic region (approximately 45 kDa) (Newton, 1995). It regulates the functions of the target protein *in vivo* by phosphorylating the hydroxyl groups and adding a phosphate group to the serine/threonine side chains (Lim et al., 2015). PKC can be divided into three subtypes according to its structural motifs and activation conditions (Keenan et al., 1997). It plays an important role in apoptosis and immune gene expression in mammals (Newton, 2018).

## Functions of phosphorylated plant viral protein

**FIGURE 3:**
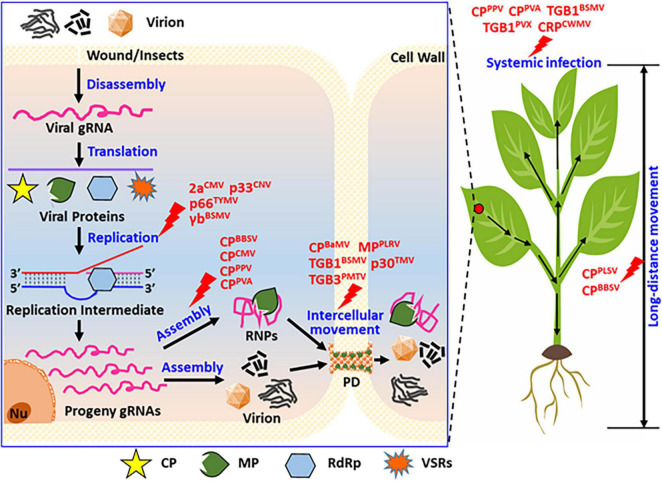
Summary of the functions of phosphorylated proteins encoded by positive single-strand RNA viruses during their life cycle. In view of the entire cell and whole plants, viral protein phosphorylation affects the replication, virion assembly, intracellular movement, long-distance movement, and systemic infection of the plants. As displayed, phosphorylation modification affected virus replication, for instance, the replicase of CMV (2a), CNV (p33), TYMV (p66), and the suppressor of BSMV (γb), these proteins not all are replicase, also contains virus-encoded RNA silencing suppressor. Phosphorylation modification of CP affected virion assemblies, such as the coat protein of BBSV, CMV, PPV, and PVA. Phosphorylation modification of viral proteins affected virus intercellular movement, such as movement protein of PLRV (MP), BSMV (TGB1), TMV (p30), PMTV (TGB3), also contains the coat protein of BaMV (CP). Phosphorylation modification of viral proteins affected virus long-distance movement, such as coat protein of PLSV (CP*^PLSV^*) and BBSV (CP*^BBSV^*). Phosphorylation modification of viral proteins affected virus systemic infection of host plant, such as coat protein of PPV (CP*^PPV^*), PVA (CP*^PVA^*), and movement protein of BSMV (TGB1*^BSMV^*), PVX (TGB1*^PVX^*), and the cysteine-rich protein of CWMV (CRP*^CWMV^*). These viruses display rod-shaped, filamentous, and spherical particles under electron-microscopy.

**FIGURE 4:**
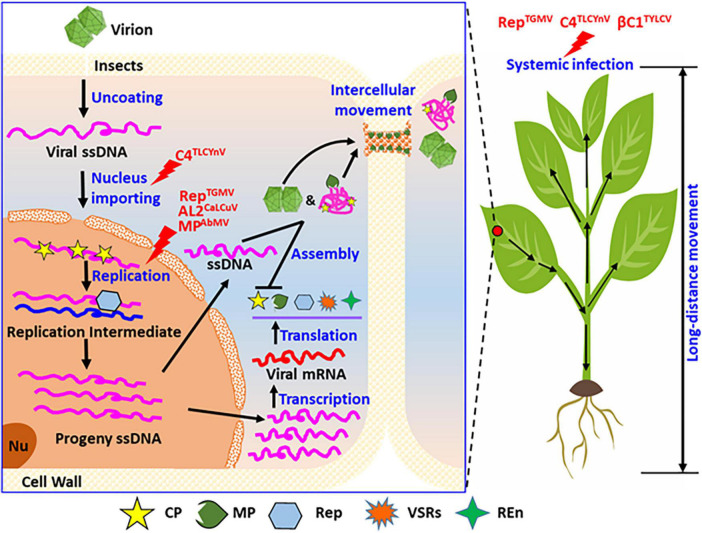
Summary of the phosphorylation functions of proteins encoded by geminiviruses during their life cycle. Considering the entire cell and whole plants, phosphorylation modification of the viral proteins regulates the different infection steps in the life cycle of geminiviruses in host plants, including the nuclear import, genomic DNA replication, and systemic infection. As shown, phosphorylation modification of C4 (C4*^TLCYnV^*) affected virus nucleocytoplasmic shuttling. Phosphorylation modification also affects the virus replication, for instance, replicase of TGMV (Rep*^TGMV^*), AL2 of CaLCuV (AL2*^CaLCuV^*), and MP of AbMV (MP*^AbmV^*). Phosphorylation modification also affects virus systemic infection of host plant, such as replicase of TGMV (Rep*^TGMV^*), C4 of TLCYnV (C4*^TLCYnV^*), and βC1 of TYLCV (βC1*^TYLCV^*). REn, replication enhancer; PD, plasmodesmata; Nu, nucleolus.

An increasing number of recent studies have revealed that plant viral proteins, including coat protein (CP), movement protein (MP), RNA-dependent RNA polymerase (RdRp), and viral suppressors of RNA silencing (VSRs), undergo phosphorylation during viral infection. The phosphorylation state of viral proteins is important for the stable infection of the virus in the host plant. *In vivo*, phosphorylation can regulate the interaction between large and small subunits of replicase, viral genomic RNA or DNA catalytic synthesis activity of replicase, stability of the viral replication complex (VRC), the interaction between viral proteins and host factors, and the binding of viral protein to RNA/DNA. This study summarizes the research on the biological functions of phosphorylated plant viral proteins (Figures 3, 4) from the perspective of the life cycle of plant viruses in plant viruses. Our research provides a reference for further plant viral protein phosphoproteomics research and provides a theoretical basis for plant viral disease prevention and control.

### Effects of phosphorylation on viral replication and transcription

*Cucumber necrosis virus* (CNV) belongs to the genus *Tombusvirus* (Alam et al., 2021). The p33 protein of CNV (p33*^CNV^*) can be phosphorylated by membrane-related kinases and PKC. The phosphorylation sites of p33*^CNV^* are Thr-205, Ser-210, and Thr-211. The replicase cofactor function of p33*^CNV^* is completely lost in plant protoplasts and yeast when p33 is mutated to a phosphorylation mimic (T/S to D). Contrastingly, when p33 was mutated to the state of dephosphorylation (T/S to A), replication can occur in plant protoplasts and yeast could occur, but the viral infection efficiency is lower than that of the wild-type virus. These results indicate that in the process of viral infection, the alanine mutants of p33*^CNV^* in the viral genome affected the synthesis of subgenomic RNA and reduced the proportions of positive- and negative-strand RNA, ultimately affecting viral replication (Figure 3, Replication). Phosphorylated p33*^CNV^* attenuates the RNA-binding ability and RdRp activity in plants (Stork et al., 2005).

*Turnip yellow mosaic virus* (TYMV) is a member of *Tymovirus* (Ni et al., 2017). The ORF1 of TYMV (ORF1*^TYMV^*) encodes an RdRp with a mass of 206 kDa, which functions in viral RNA replication. *In vivo*, ORF1*^TYMV^* produces 140 kDa (p140) and 66 kDa (p66) replicase subunits via self-cleavage. The 66 kDa replicase subunits can be phosphorylated in plants, three main phosphorylation sites, thr-64, Ser-80, and Ser-326, have been identified via mass spectrometry. Dephosphorylation or phosphorylation-mimic mutants at the three phosphorylation sites of the 66 kDa replicase subunit have different effects on viral infectivity. The double phosphorylation-mimic mutant p66*^T64D/S80D^* and the single mutants p66*^S326A^*, p66*^S326D^*, and p66*^S326T^* have remarkable effects on viral infectivity, thereby significantly reducing the viral genomic RNA and CP protein accumulation levels. Phosphorylation of p66 does not affect its interaction with p140. In the viral infection cycle, the p66 phosphorylation is inhibited by the interactions between p140 and p66 subunits. These interactions mark the initiation of the viral replication complex assembly at the host cell membrane. Ser-326 site of p66 is the key to viral RNA synthesis regulation, and the phosphorylation/dephosphorylation of p66*^S326^* affects the binding and depolymerization of viral genomic RNA and the replication complex. These results indicate that the phosphorylation of p66 is a molecular switch that regulates the speed of viral replication speed in a self-optimized manner and balances the interaction between the virus and the host plant. The speed of adaptive viral genomic RNA replication could guarantee the long-term coexistence of the virus in the host plant in terms of evolution (Jakubiec et al., 2006; Jakubiec and Jupin, 2007; Figure 3, Replication).

*Barley stripe mosaic virus* (BSMV) γb protein is a typical VSR and viral symptom determinant (Bragg et al., 2004). The γb*^BSMV^* can be phosphorylated by PKA-like protein kinase in *N. benthamiana in vitro* and *in vivo*. Ser-96 is the main phosphorylation site of γb in plants. The γb*^S96A^* mutant has reduced local and systemic VSR activities due to the compromised 21-bp dsRNA binding activity and causes necrosis of infected leaves. Overexpression of other VSRs *in trans* or *cis* cannot rescue the necrosis induced by the BSMV γb*^S96A^* mutant. These results demonstrated that suppression of cell death via γb phosphorylation at Ser-96 is functionally distinct from the RNA silencing suppressor activity. In addition, the serine/threonine/tyrosine kinase (NbSYT46) phosphorylates and interacts with γb. The antiviral role of NbSTY46 involves kinase activity, accordingly, the NbSTY46*^T436A^* mutant lacking kinase activity not only loses the ability to phosphorylate or interact with γb but also fails to sustain virus systemic infection in plants. Self-phosphorylated NbSYT46 directly interacts with and phosphorylates the γb protein, which negatively regulates the viral replication and inhibits viral infection (Zhang et al., 2018, 2021; Figure 3, Replication).

*Barley yellow striate mosaic virus* (BYSMV) is a member of the genus *Cytorhabdovirus*, which elicits chlorotic striate and mosaic symptoms in the leaves of cereal plant species (Cao et al., 2018). BYSMV genome is a long, negative, single-stranded RNA (Yan et al., 2015). In BYSMV infection cycle, the phosphoprotein (P) in barley has two forms, with different migration rates corresponding to 42 kDa (p42) and 44 kDa (p44). Mass-spectrometry identified five C-terminal serine-rich regions (SR) of the phosphoprotein (^189^SASRPSSIAS^198^) that were phosphorylated. The viral dephosphorylation mutant (BYSMV-P*^S5A^*) enhanced viral replication, whereas the transcription was facilitated by the phosphorylation-mimic mutant (BYSMV-P*^S5D^*). *In vivo*, the two forms of P proteins (P*^S5A^* and P*^S5D^*) were preferentially associated with the nucleocapsid protein-RNA template and large polymerase protein, providing optimal replication and transcription of BYSMV, respectively. Biochemical assays demonstrated that plant and insect CK1 proteins can phosphorylate the SR motif of the P protein. Superphosphorylation of SR induces conformational changes in proteins. Moreover, the overexpression of CK1 or a dominant-negative mutant impairs the phosphorylation/dephosphorylation balance between p42 and p44, thereby compromising viral infection in barley. These observations indicate that BYSMV recruits the conserved CK1 kinases to finely regulate the replication-transcription phase during the viral life cycle and achieve optimal cross-kingdom infection of plants and insect vectors (Gao et al., 2020).

Ding et al. (2022) demonstrated that the barley (*Hordeum vulgare*) MAPK-MPK3 (HvMPK3) and the planthopper ERK (LsERK) proteins can interact with the viral structural protein nucleoprotein and directly phosphorylate this N protein at Ser-290. Overexpression of HvMPK3 could inhibit BYSMV infection, and barley plants treated with the MAPK pathway inhibitor U0126 are more susceptible to BYSMV. The dsRNA was synthesized and microinjected into the planthopper to knock down the *LsERK*, then the virus infection was promoted. A phosphomimetic mutant of the nucleoprotein Ser-290 (S290D) completely abolished virus infection because of the impaired self-interaction of BYSMV N and the formation of unstable N–RNA complexes. These results demonstrated that the conserved MAPK and ERK directly phosphorylate the viral nucleoprotein to trigger immunity against cross-kingdom immunity to BYSMV in host plants and its insect vectors (Ding et al., 2022).

### Effect of phosphorylation on the packaging of viral particles

*Beet black scorch virus* (BBSV) belongs to the genus *Necrovirus* in the family *Tombusviridae* (Xu et al., 2016). CP*^BBSV^* can be phosphorylated at Thr-41 by the cAMP-dependent protein kinase (PKA)-like kinases *in vivo* and *in vitro*. The non-phosphorylated state of CP*^T41^* does not affect the initial viral RNA replication stage, however, viral genomic RNA accumulation is affected as the infection progresses. CP*^BBSV^* phosphorylation is inferred to be involved in the later stages of genomic RNA replication, effectively interfering with the host defense system. RNase-sensitivity assays revealed that the BBSV*^T41A^* and BBSV*^T41E^* mutant viruses are easily degraded by endogenous ribonucleases in *Nicotiana benthamiana*. Electron microscopy revealed that the BBSV*^T41A^* and BBSV*^T41E^* mutants are abnormal compared to the wild-type BBSV particle (Figure 3, Virion Assembly). These findings indicated that the CP*^T41^* mutant could not package viral RNAs into intact viral particles to protect against genomic RNA degradation. The phosphorylation state of CP*^T41^* can regulate the virus particles in plants, thereby finely regulating BBSV infection in plants (Zhao et al., 2015).

### Effect of phosphorylation on viral movement

*Bamboo mosaic virus* (BaMV) is a member of the genus *Potexvirus* and possesses a positive single-stranded RNA genome (Chen et al., 2017). The CP is phosphorylated by CK2 in *N. benthamiana* (NbCK2). Studies revealed that the main phosphorylation site is Ser-241 and both CP*^BaMV^* and NbCK2α subunits are colocalized on the plasmodesmata (PD). These results suggest that phosphorylation of CP*^BaMV^* may be involved in the viral cell-to-cell movement. Although *in vitro* binding assays showed that the RNA-binding ability of CPS^241A^ mutant was not impaired, the cell-to-cell movement ability of mutant BaMV-CPS^241A^ was reduced. If the Ser-241 site is mutated to D (phosphorylation mimics, CP^ S241D^), its RNA-binding ability is significantly weakened, and the cell-to-cell movement ability of the corresponding mutant virus is blocked. Further deletion of the AA from 240 to 242 in CP*^BaMV^* enhances the RNA-binding ability of CP, but the cell-to-cell movement ability of the corresponding mutant virus is affected. These results indicate that the NbCK2 regulates the RNA-binding ability of CP*^BaMV^* by phosphorylating the AA 241 (Hung et al., 2014). The p20 protein, encoded by BaMV satellite RNA (satBaMV), can also be phosphorylated in plants. *In vitro* experiments showed that p20 can be phosphorylated via a total intracellular protein extraction and the Ser-11 is the phosphorylation site. The p20*^S11D^* mutant almost completely loses its ability to bind to wild-type p20 and mutant p20*^S11A^ in vitro* (Vijayapalani et al., 2012; Figure 3, Intercellular Movement).

*Potato mop-top virus* (PMTV), belongs to the genus of *Pomovirus* and the family *Virgaviridae* (Cowan et al., 2018). Genomic RNA3 of PMTV encodes TGB3, which is one of the three movement proteins. TGB3*^PMTV^* participates in viral intracellular movement in plants. Studies have found that the Tyr-87, Tyr-88, Tyr-89, and Tyr-120 are phosphorylated in TGB3. *In vitro* phosphorylation experiments have shown that the phosphorylation signal of TGB3 is absent when the tyrosine (Y) is mutated to alanine (A). These results demonstrated that the Tyr (87—89, 120) is the actual phosphorylation site of TGB3.

In addition, the phosphorylation/dephosphorylation state of TGB3 affects the intensity of its interaction with TGB2. Mutation of the tyrosine to alanine at positions 87–89 of TGB3*^PMTV^* enhances the interaction intensity between TGB3 and TGB2 in yeast. However, the intercellular movement ability of the corresponding viral mutant (PMTV-TGB3*^T87–89A^*) is weakened in *N. benthamiana*. Although the TGB3*^Y120A^* mutant does not affect the interaction with TGB2 protein, it cannot infect *N. benthamiana*. This demonstrates that tyrosine phosphorylation modification exists in plant viral proteins and is also a method of regulating plant virus movement and infectivity (Figure 3, Intercellular Movement; Samuilova et al., 2013).

*Barley stripe mosaic virus* (BSMV) is a representative species of *hordeivirus* (Wang et al., 2021). The triple gene blocks 1 (TGB1) protein is one of the three movement proteins encoded by BSMV. *In vitro* phosphorylation experiments showed that TGB1*^BSMV^* can be phosphorylated by CK2 kinase in the *N. benthamiana* and barley. Further research revealed that the Thr-401 is the main phosphorylation site, and Thr-395 is the anchor site of CK2 *in vivo*. Mutations in Thr-395 and Thr-401 mimic the dephosphorylation status (T395A, T395E, T401A, and T401E). The mutant viruses can systematically infect barley and wheat, however, the infection efficiency and viral accumulation levels decreased dramatically. In *N. benthamiana*, only the mutant virus BSMV-TGB1*^T395A^* can establish a systemic infection. Together, these results demonstrate that the phosphorylation/dephosphorylation status of TGB1 at Thr-395 and Thr-401 affects the viral systemic movement ability differently in monocot and dicot hosts. This may be a consequence of the architecture of host-specific vasculature. Furthermore, the phosphorylation of TGB1 promotes viral infection by enhancing the interaction intensity with TGB3 proteins, which is apt to form the viral ribonucleoprotein complex (BSMV intracellular movement complex). However, the phosphorylation modification of TGB1 is not sufficient to substantially alter the TGB localization patterns visualized via laser confocal microscopy (Figure 3, Intercellular Movement; Hu et al., 2015).

### Effects of phosphorylation on viral infectivity and pathogenicity

*Chinese wheat mosaic virus* (CWMV), a member of the genus *Furovirus* and family of *Virgaviridae*, is an RNA virus with two positive-sense single-stranded genomic RNAs (Yang et al., 2020). Ser-162 and Ser-165 of the CWMV cysteine-rich protein (CRP*^CWMV^*) are the two phosphorylation sites catalyzed by wheat SAPK7 protein *in vivo* and *in vitro*. Mutational analyses have shown that the double-site phosphorylation-mimic mutant virus (CWMV-CRP*^S162/165D^*) enhances viral infectivity in plants because of the suppressed cell death activity and hydrogen peroxide production by the CRP*^S162/165D^* protein. Furthermore, the CWMV-CRP*^S162/165D^* interacts with the RNA-binding protein UBP1-associated protein 2C (TaUBA2C) in wheat, whereas the phosphorylation-deficient mutant virus (CWMV-CRP*^S162/165A^*) does not. Silencing of TaUBA2C promotes viral infection in wheat plants, while the overexpression of TaUBA2C inhibits viral infection. TaUBA2C recruits the pre-mRNAs of TaNPR1, TaPR1, and TaRBOHD to induce plant cell death and hydrogen peroxide production, ultimately inhibiting viral infection. This effect can be suppressed by phosphorylated CRP*^S162/165D^* through direct binding of TaUBA2C, resulting in changes in the chromatin-bound status and the attenuation of the RNA- or DNA-binding activity of TaUBA2C in plants (Figure 3, Systemic Infection; Li et al., 2022).

*Tomato yellow leaf curl virus* (TYLCV) is a member of *Geminivirus* with a single genomic component that is composed of a single-stranded circular DNA (DNA-A) and satellite DNA (DNA-B) (Prasad et al., 2020). The βC1 protein is encoded by satellite DNA-B, which functions as a VSR and a symptom determinant (Hu et al., 2019). βC1*^TYLCV^* interacts with a variety of host factors that interfere with multiple antiviral immune pathways in plants. An *in vitro* yeast two-hybrid assay (Y2H) and *in vivo* bimolecular fluorescence complementation (BiFC) revealed that βC1*^TYLCV^* interacts with tomato SNFI-related kinase (SnRK1). Phosphorylation experiments showed that βC1*^TYLCV^* is phosphorylated by SnRK1 kinase, and Ser-33, Thr-78, Tyr-5, and Tyr-110 are the main phosphorylation sites. The symptoms of phosphorylation-deficient mutant viruses (TYLCV-βC1^*S*33*A*^, -βC1^*T*78*A*^, -βC1^*S*33*A*^, and -βC1^*T*78*A*^) are aggravated, and the viral genomic DNA accumulation levels are significantly higher than those of the wild-type virus. The phosphorylation-mimic mutant viruses (TYLCV-βC1^*S*33*D*^, -βC1^*T*78*D*^, and -βC1^*S*33*D*/*T*78*D*^) delay infection, and the viral DNA accumulation levels are completely opposed to those in the phosphorylation-deficient mutant viruses. These results indicate that SnRK1 interacts with and phosphorylates βC1*^TYLCV^* protein to weaken viral infectivity in tomatoes. Compared with those of the double mutant virus TYLCV-βC1-2D infection, the symptoms of TYLCV-βC1-2D/2E (S33D/T78D/Y5E/Y110E) infection are further attenuated, whereas the VSR activity of βC1-2D/2E is significantly weakened. Furthermore, phosphorylation of the βC1 protein by SnRK1 greatly affects its interaction with *N. benthamiana* ASYMMETRIC LEAVES 1, and regulates the induction of symptoms (Figure 4, Systemic Infection; Shen et al., 2011; Zhong et al., 2017).

*Yunnan tomato leaf curl virus* (TLCYnV) is a member of the *Geminivirus* family with a single-component genomic DNA (Mei et al., 2020). The C4 protein of TLCYnV (C4*^TLCYnV^*) is a symptom determinant in plants (Mei et al., 2018b). *In vivo* and *in vitro* phosphorylation experiments demonstrated that C4*^TLCYnV^* is phosphorylated by the host NbSKη, and the Thr-51 is the corresponding phosphorylation site. Phosphorylation of C4*^TLCYnV^* plays a key role in plant viral pathogenicity. The pathogenicity and viral genomic DNA accumulation levels of the dephosphorylation mutant (TLCYnV-C4*^T51A^*) are significantly reduced. Furthermore, subcellular localization revealed that the C4*^T51A^* mutant confines the NbSKη protein to the cell membrane, and myristoylation of the C4 N-terminal Gly-2 position directly results in the cell membrane localization. The pathogenicity and viral genomic DNA accumulation levels of the mutant TLCYnV-C4*^G2A^* virus are significantly reduced, respectively. During TLCYnV-C4*^G2A^* virus infection, NbSKη cannot localize to the cell membrane. Chemical inhibition of *N*-myristoyltransferases or exportin-α enhances the nuclear retention of C4*^TLCYnV^*, and mutations in the putative phosphorylation (T51A) or myristoylation sites (G2A) of C4*^TLCYnV^* result in increased nuclear localization and reduced severity of the C4-induced developmental abnormalities. These results suggest that the nucleocytoplasmic shuttling of C4*^TLCYnV^* via protein modifications, including NbSKη-mediated protein phosphorylation, protein myristoylation, and the interaction with exportin-α, is critical for viral pathogenicity in plants (Figure 4, Systemic Infection; Mei et al., 2018a).

Table 2 shows several plant virus-encoded proteins that can be phosphorylated. Single viral protein phosphorylation often changes the biological characteristics of the protein itself and can affect the viral infection cycle in plants. The host CK2 is not only involved in plant growth and development but also regulates the function of a variety of viral proteins through phosphorylation. The virus often achieves maximum replication during co-evolution through an interaction balance between host defense and survival. Phosphorylation modifications often act as a molecular switch that guarantees optimal viral replication and prolonged viral existence in virus-specific hosts.

**TABLE 2:** Phosphorylation modifications of plant virus proteins.

Regulatory functions	Virus taxonomy	Viral protein	Phosphorylation kinase	Phosphorylation site	References
Assembly of virus particles	ss(+)RNA	CP*^BBSV^*	PKA	Thr-41	Zhao et al., 2015
		CP*^CMV^*	Cellular kinases	Ser-148	Nemes et al., 2019
		CP*^PVA^*	CK2	Thr-242	Ivanov et al., 2001; Ivanov et al., 2003
		CP*^PPV^*	CK2	Ser-25, Ser-81, Ser-101, Ser-118	Martínez-Turiño et al., 2018
Cell-to-cell movement	ss(+)RNA	CP*^BaMV^*	CK2	Ser-241	Hung et al., 2014
		TGB1*^BSMV^*	CK2	Thr-401, Thr-395	Hu et al., 2015
		MP17*^PLRV^*	PKC-like kinase	Ser-71, Ser-79, Ser-137, Ser-140	Link et al., 2011
		TGB3*^PMTV^*	Cellular kinases	Tyr-87,Tyr-88,Tyr-89, Tyr-120	Samuilova et al., 2013
		P30*^TMV^*	CK2, PAPK1	Ser-258, Thr-261, Ser-264	Trutnyeva et al., 2005; Waigmann et al., 2000
Long-distance movement	ss(+)RNA	CP*^BBSV^*	PKA	Thr-41	Zhao et al., 2015
		TGB1*^PSLV^*	CK1, PKA, PKC	Unknown	Makarov et al., 2012
Interaction with other proteins	ss(+)RNA	CP*^BBSV^*	Unknown	Tyr-194	Gao et al., 2022
		TGB3*^PMTV^*	Cellular kinases	Tyr-87, Tyr-88, Tyr-89, Tyr-120	Samuilova et al., 2013
		2a*^CMV^*	Cellular kinases	Unknown	Kim et al., 2002
	ssDNA	C4*^TLCYnV^*	NbSKη	Thr-51	Mei et al., 2018a
		βC1*^TYLCV^*	SnRK1	Ser-33, Thr-78, Tyr-5, Tyr-110	Zhong et al., 2017; Shen et al., 2011
Self-interaction	ss(+)RNA	TGB1*^PSLV^*	CK1, PKA, PKC	Unknown	Makarov et al., 2012
		P20*^satBaMV^*	Cellular kinases	Ser-11	Vijayapalani et al., 2012
	ss(-)RNA	NP*^BYSMV^*	HvMP3, LsERK	Ser-290	Ding et al., 2022
Subcellular localization	ss(+)RNA	MP*^ToMV^*	CK2, NtRIO	Ser-37, Ser-238,	Matsushita et al., 2003; Kawakami et al., 1999; Yoshioka et al., 2004
		2b*^CMV^*	CK2	Ser-40, Ser-42	Nemes et al., 2017
	ssDNA	C4*^TLCYnV^*	NbSKη	Thr-51	Mei et al., 2018a
Viral infectivity and pathogenicity	ss(+)RNA	CP*^BBSV^*	Unknown	Tyr-194	Gao et al., 2022
	dsDNA	CP44*^CaMV^*	CK2	Ser-66, Ser-68, Ser-72	Champagne et al., 2007; Chapdelaine et al., 2002
	ssDNA	MP(βC1)*^AbMV^*	CK2	Thr-221, Ser-223, Ser-250	Kleinow et al., 2008; Kleinow et al., 2009
	ss(+)RNA	TGB1*^BSMV^*	CK2	Thr-401, Thr-395	Hu et al., 2015
		TGB3*^PMTV^*	Cellular kinases	Tyr-87, Tyr-88, Tyr-89, Tyr-120	Samuilova et al., 2013
		TGBp1*^PVX^*	CK2-like kinase	Ser-165	Módena et al., 2008
		γb*^BSMV^*	PKA, NbSTY46	Ser-96	Zhang et al., 2018; Zhang et al., 2021
		CRP*^CWMV^*	TaSAPK7	Ser-162, Ser-165,	Li et al., 2022
	ssDNA	βC1*^TYLCV^*	SnRK1	Ser-33, Thr-78, Tyr-5, Tyr-110	Zhong et al., 2017; Shen et al., 2011
		C4*^TLCYnV^*	NbSKη	Thr-51	Mei et al., 2018a
		Rep*^TGMV^*	SnRK1	Ser-97	Shen et al., 2018
Virus replication and transcription	ss(+)RNA	CP*^PVX^*	CK1, CK2	Unknown	Atabekov et al., 2001
		2a*^CMV^*	Cellular kinases	Unknown	Kim et al., 2002
		P33*^CNV^*	Cellular kinases	Thr-205, Ser-210, Thr-211	Stork et al., 2005
		66 kDa RDRP*^TYMV^*	Cellular kinases	Thr-64, Ser-80, Ser-326	Jakubiec and Jupin, 2007; Jakubiec et al., 2006
		γb*^BSMV^*	PKA, NbSTY46	Ser-96	Zhang et al., 2018; Zhang et al., 2021
	ss(–)RNA	P*^BYSMV^*	CK1	Ser-189, Ser-191, Ser-194, Ser-195, Ser-198	Gao et al., 2020
		NP*^BYSMV^*	HvMP3, LsERK	Ser-290	Ding et al., 2022
	ssDNA	AL2*^CaLCuV^*	SnRK1	Ser-109	Shen et al., 2014

## Conclusion and perspectives

As one of the most extensive PTMs, phosphorylation has now reached the stage where it is significant in almost every physiological event and plays important roles in regulating multiple biological activities, including cell signal transduction, protein–protein interactions, PTM-crosstalk, self-stability, and subcellular localization (Figure 1). Investigations of protein phosphorylation modification are important for revealing the molecular mechanisms underlying interactions between plant defense and viral counterdefense. Here, we summarized the current phosphorylation detection methods for plant viral proteins. Using the combination of *in vivo* and *in vitro* phosphorylation experiments, it is easy to determine the detailed phosphorylation sites of viral protein and host kinases. Various studies have revealed that viral proteins often act as molecular switches that display functions according to the two statuses, phosphorylation and dephosphorylation, promoting or preventing infection in a particular host. The different statuses of viral proteins are catalyzed by host kinases, including CK1, CK2, GSK3, PKA, and PKC. Table 2 summarizes the common phosphorylation motifs for the substrates of different kinases and provides a reference for identifying the phosphorylation sites of target viral proteins and their corresponding host kinases. CK2 can phosphorylate the CP and MP of different plant viruses, and the function of CK2 has been widely studied in relation to plant–virus interactions. These findings suggest that CK2 plays an essential role in the regulation of the viral infection cycle. However, it is unclear how the same kinase phosphorylates MP and CP, and how they coordinate with each other to benefit virus infection. Our study reviewed the methods for identifying phosphorylation and the biological functions of phosphorylated viral proteins reported to date. After phosphorylation in plant cells, the subcellular localization, pathogenicity, self-interactions, and interactions with other viral proteins are altered, which finally regulates virus replication and transcription during the infection process (Figure 1). The summaries provide a reference and direction for further research into how viral protein phosphorylation balances the continuous viral infection and the host’s moderate response and the defense or counter-defense role of plant kinases in the interactions between the plant virus and host.

In addition to phosphorylation, the side chain hydroxyl group on the serine and threonine residues of the target protein often undergo *O*-GlcNAcylation in eukaryote. The *O*-GlcNAcylation modification is catalyzed and removed by the *N*-acetylglucosamine transferase (OGT) and the O-linked *N*-acetylglucosamine hydrolase (OGA), respectively (Yang and Qian, 2017). The *O*-GlcNAcylation modification can regulate various basic cellular processes, such as transcription (Golks et al., 2007), epigenetic modification (Hanover et al., 2012; Dehennaut et al., 2014; Lewis and Hanover, 2014; Singh et al., 2015), intracellular signaling transduction (Vosseller et al., 2002; Zhang K. et al., 2014; Xu et al., 2019), and virus infection (Chen et al., 2005; Kim et al., 2011; Pérez et al., 2013; Song et al., 2019; Hu et al., 2021). The same serine and threonine target sites determine the interactions between *O*-GlcNAcylation and phosphorylation *in vivo*. Many studies have shown that in various proteins, *O*-GlcNAcylation often occurs with reciprocal or sequential phosphorylation of the same or neighboring residues (Hart et al., 2007, 2011). *O*-GlcNAcylation can temporally regulate the insulin signaling pathways in humans (Zhang K. et al., 2014). ATK is a type of serine/threonine kinases that are involved in cell survival and metabolism (Vosseller et al., 2002). The *O*-GlcNAcylation and phosphorylation sites of AKT were mapped to the same positions (Thr-308 and Ser-473) (Shi et al., 2015). These observations demonstrate that *O*-GlcNAcylation of AKT kinases directly suppresses its phosphorylation, which shows a “Yin-Yang” model with antagonistic effects on specific AAs of particular proteins (Butkinaree et al., 2010). In winter wheat verbalizations, the possible correlation between the modified sites and the computationally predicted structures of the 31 proteins containing both the *O*-GlcNAcylation and phosphorylation modifications were explored, and their result showed that they could be divided into two major patterns: competitive (Yin-Yang model) and coexisting (coordinately regulated) (Xu et al., 2019). In plant virology studies, CP*^PPV^* was found to be modified via *O*-GlcNAcylation during virus infection, which promoted the stability of the CP protein and affected the virus movement and replication (Chen et al., 2005; Kim et al., 2011; Pérez et al., 2013). Seven AA sites of the CP*^PPV^*, including the Thr-19, Thr-21, Thr-24, Thr-26, Thr-41, Thr-53, and Ser-65, show *O*-GlcNAcylation modifications during PPV infection (Pérez et al., 2013), whereas Ser-25, Ser-81, Ser-101, and Ser-118 were phosphorylated (Martínez-Turiño et al., 2018). The correlation between the Ser-25 phosphorylation and *O*-GlcNAcylation of Thr-19, Thr-21, Thr-24, and Thr-26 in the CP*^PPV^* needs further investigation. Exploration of the coexisting or competitive patterns of these two major modifications on neighboring residues (AA 19-26) of CP*^PPV^* has profound significance for a deeper understanding of the plant virus life cycle.

More than 20 viral proteins are phosphorylated during viral infection cycle (Figures 3, 4). Do other plant viral proteins also undergo phosphorylation modification with different regulatory effects on viral infection? A variety of kinases in plants have been identified in plants, with less than 10 types found to be directly involved in the phosphorylation. Nevertheless, the types and functions of kinases involved in the phosphorylation of viral proteins require further exploration. This study revealed that only the CK2 and PKA can phosphorylate various plant viral proteins. Are other viral proteins phosphorylated by these two kinases? Can different viral proteins encoded by the same virus be phosphorylated by the same host kinase? We identified numerous phosphorylation sites of plant viral proteins via mass spectrometry, especially in target proteins that can be phosphorylated via total protein extractions *in vitro*. The finding is not representative of the *in vivo* conditions because the total protein obtained from plant contained many kinases, and phosphorylation can occur between the kinase and substrate that would not normally be in contact *in vivo*. Different protein kinases can phosphorylate multiple sites of the same and different viral proteins encoded by the same virus, and these modifications have different regulatory roles in plant–virus interactions.

In plant virology, the core question is how to coordinate plant resistance with yield and quality. The phosphorylation studies discussed here are promising. Massive progress in the identification of the major substrates of each protein kinase and phosphatase has been achieved in recent years using the modern MS-based proteomics. However, major gaps in our knowledge still exist regarding the identities of the key substances and protein kinases, and how their phosphorylation contributes to changes in cell physiology in response to particular stimuli. A certain genome size determines a finite number of kinases and phosphatase in plants, and there are multiple potential phosphorylation sites within a single protein, which implies that the kinase and phosphatase isoforms may have overlapping specificities. These kinases are involved in the phosphorylation of several hundreds of substrates *in vivo*. In addition, the substrate of the target kinase is often cell-specific. Explaining the distinctive effects of various stimuli on different tissues requires further consideration. Hence, powerful methods, coupled with further exploitation of important methodological and technical advances, are needed to identify the substance protein and the corresponding target kinase. Of greater importance is research to control the outbreaks and devastation caused by plant viruses, develop tissue and phosphor-specific antibodies, certain cell-type-specific permeant inhibitors of protein kinase, and design new therapies via taking advantage of the distinct cell types not expressing a particular protein kinase or phosphatase.

In future plant–virus protein phosphorylation studies, the phosphorylation sites on viral proteins can be identified in detail by mass spectrometry combined with the *in vitro* phosphorylation experiments. The corresponding kinases can also be identified in the follow-up research using reverse genetics techniques. Furthermore, it would be beneficial to explore the impact of certain types of kinase and viral mechanism, including viral replication, viral transcription, and the host’s defense response. However, several proteins encoded by one certain plant virus can be phosphorylated by several types of kinases. The elucidation of specific kinases and their substrates and determination of the corresponding relationship are essential aspects that should be considered in future studies of biological function. If the common pathways involved in the broad-spectrum antiviral immunity conferred by kinases can be identified, we could cultivate new antiviral crop germplasm through transgenic or CRISPR-Cas9-mediated gene editing methods. Moreover, small-molecule activators or inhibitors could be designed according to the advanced structures of target kinases, which could be used as novel pesticides for spraying crop leaves in the field for virus control.

## Author contributions

KZ and XZ contributed substantially to the conception and design of this review article and co-wrote the manuscript. KZ and ZH reviewed the manuscript before submission for its intellectual content. All authors gave final approval of the published version.

## Conflict of interest

The authors declare that the research was conducted in the absence of any commercial or financial relationships that could be construed as a potential conflict of interest.

## Publisher’s note

All claims expressed in this article are solely those of the authors and do not necessarily represent those of their affiliated organizations, or those of the publisher, the editors and the reviewers. Any product that may be evaluated in this article, or claim that may be made by its manufacturer, is not guaranteed or endorsed by the publisher.

## Footnotes

1
http://kinasephos2.mbc.nctu.edu.tw

2
http://www.cbs.dtu.dk/services/NetPhos/

## Abbreviations

AbMV: abutilon mosaic virus
BaMV: bamboo mosaic virus
BBSV: beet black scorch virus
BSMV: barley mosaic stripe virus
CaLCuV: cabbage leaf curl virus
CMV: cucumber mosaic virus
CNV: cucumber necrosis virus
CWMV: Chinese wheat mosaic virus
CP: coat protein
MP: movement protein
Nu: nucleolus
PD: plasmodesmata
PLRV: potato leaf roll virus
PMTV: potato mop-top virus
PPV: plum pox virus
PSLV: poa semilatent virus
PTM: post-translational modifications
PVA: potato virus A
PVX: potato virus X
TLCYnV: Yunnan tomato leaf curl virus
TGMV: tomato golden mosaic virus
CaLCuV: cabbage leaf curl virus
TYLCV: tomato yellow leaf curl virus
RdRp: RNA-dependent RNA polymerase
Ren: replication enhancer
Rep: replicase
RNPs: ribonucleoprotein
TMV: tobacco mosaic virus
TYMV: turnip yellow mosaic virus
VSR: virus-encoded RNA silencing suppressors
REn: replication enhancer.
